# Best treatment options for occult breast cancer: A meta-analysis

**DOI:** 10.3389/fonc.2023.1051232

**Published:** 2023-05-12

**Authors:** Rong Wang, Hong-xin Yang, Jie Chen, Jian-jun Huang, Qing Lv

**Affiliations:** ^1^ Department of Breast Surgery, West China Hospital, Sichuan University, Chengdu, Sichuan, China; ^2^ Department of Breast Surgery, Affiliated Hospital of Guizhou Medical University, Guiyang, Guizhou, China; ^3^ Department of General Surgery, Affiliated Hospital of Guizhou Medical University, Guiyang, Guizhou, China

**Keywords:** treatment, mortality rate, surgery, radiotherapy, occult breast cancer

## Abstract

**Objectives:**

Occult breast cancer (OBC) is a rare malignant breast tumor. Because of the rare cases and limited clinical experience, a huge therapeutic difference has existed all over the world and standardized treatments have yet been established.

**Methods:**

A meta-analysis was conducted using MEDLINE and Embase databases to identify the choice of OBC surgical procedures in all studies: (1) patients undergoing axillary lymph node dissection (ALND) or sentinel lymph node biopsy (SLNB) only; (2) patients undergoing ALND with radiotherapy (RT); (3) patients undergoing ALND with breast surgery (BS); (4) patients undergoing ALND with RT and BS; and (5) patients undergoing observation or RT only. The primary endpoints were mortality rates, the second endpoints were distant metastasis and locoregional recurrence.

**Results:**

Among the 3,476 patients, 493 (14.2%) undergo ALND or SLNB only; 632 (18.2%) undergo ALND with RT; 1483 (42.7%) undergo ALND with BS; 467 (13.4%) undergo ALND RT and BS, and 401 (11.5%) undergo observation or RT only. After comparing the multiple groups, both groups 1 and 3 have higher mortality rates than group 4 (30.7% vs. 18.6%, p < 0.0001; 25.1% vs. 18.6%, p = 0.007), and group 1 has higher mortality rates than groups 2 and 3 (30.7% vs.14.7%, p < 0.00001; 30.7 vs. 19.4%, p < 0.0001). Group (1 + 3) had a prognosis advantage over group 5 (21.4% vs. 31.0%, p < 0.00001). There was no significant difference both in the distant recurrence rates and locoregional rates between group (1 + 3) and group (2 + 4) (21.0% vs. 9.7%, p = 0.06; 12.3% vs. 6.5%, p = 0.26).

**Conclusion:**

On the basis of this meta-analysis, our study indicates that BS including modified radical mastectomy (MRM) and breast-conserving surgery (BCS) combined RT may appear as the optimal surgical approach in patients with OBC. RT cannot prolong both the time of distant metastasis and the local recurrences.

## Introduction

Breast cancer is the most commonly diagnosed cancers in the world, with the rapidly increasing incidence, affecting one in eight women in their lifetime (1, 2). Occult breast cancer (OBC) is a special type of breast cancer, and its detection rate in breast cancer is very low. The incidence of OBC varies greatly from 0.1% to 15.9% (3–5). Patients with OBC are defined as a group of people for whom the primary lesion in the breast gland cannot be found through clinical tests and imaging and the metastasis of axillary lymph nodes (ALNs) or other organs is mainly taken as the first symptom; for these patients, other metastatic sites need to be confirmed as the source of breast cancer lesions. To date, occult breast lesions cannot be found through effective breast examination, which leads to patients to often neglect to seek medical advice. Meanwhile, OBC may have a worse prognosis, as ALN status is an important prognostic indicator of survival in breast cancer, making this disease a serious threat to women’s life (6–8). The definition of OBC is still controversial, although some studies firmly assume that OBC may originate from ectopic breast tissue that is present in ALNs, there are tissues with proliferative changes in the ectopic breast in ALN, and some of them may undergo malignant transformation (9). Kuehlmann et al. (10) have reported that breast cancer lesions can be found in prophylactic mastectomy specimens or reduction mammoplasty, as many as 76.2% of patients presented benign histopathological and carcinoma diseases found in reduction mammoplasty, suggesting that, by reason of insufficient detection methods, the potential incidence of OBC is higher than expected (11–13).

The National Comprehensive Cancer Network (NCCN) recommends that patients with OBC should receive mastectomy + ALND or ALND + whole breast radiotherapy RT (14), but great differences have been identified in treatment methods and prognoses in the real world (15). Because of few studies on the tissue source and clinical research of OBC, at present, there is no unified conclusion to prove which treatment is more effective (16). We retrospectively analyzed the survival information of multiple cohort studies to evaluate the most appropriate surgery and treatment options.

## Methods

### Search strategy

In this study, a systematic review and meta-analysis of retrospective analyses of OBC was conducted on the basis of the definition of the guidelines. We searched Embase and MEDLINE (PubMed) for studies published in English using combinations of the terms “occult breast cancer”, “occult breast carcinoma”, “occult primary breast cancer”, and “occult breast neoplasm” as well as the keywords “primary axillary metastases”, “with limits”, and “human”. Relevant reviews, meta-analyses, and references cited in these papers were also checked for potential studies. At the same time, the Cochrane Database of systematic reviews and Cochrane Central Register of controlled trials were searched. We defined OBC as a carcinoma that presented with axillary metastases in the absence of a primary breast tumor on physical examination, imaging, preoperative biopsy, and postoperative pathological examination. Because most OBC patients with distant metastases may not have the opportunity to undergo a BS and they may have worse prognosis than those with early breast cancer, they were not included in this analysis.

### Selection criteria

**Figure 1:**
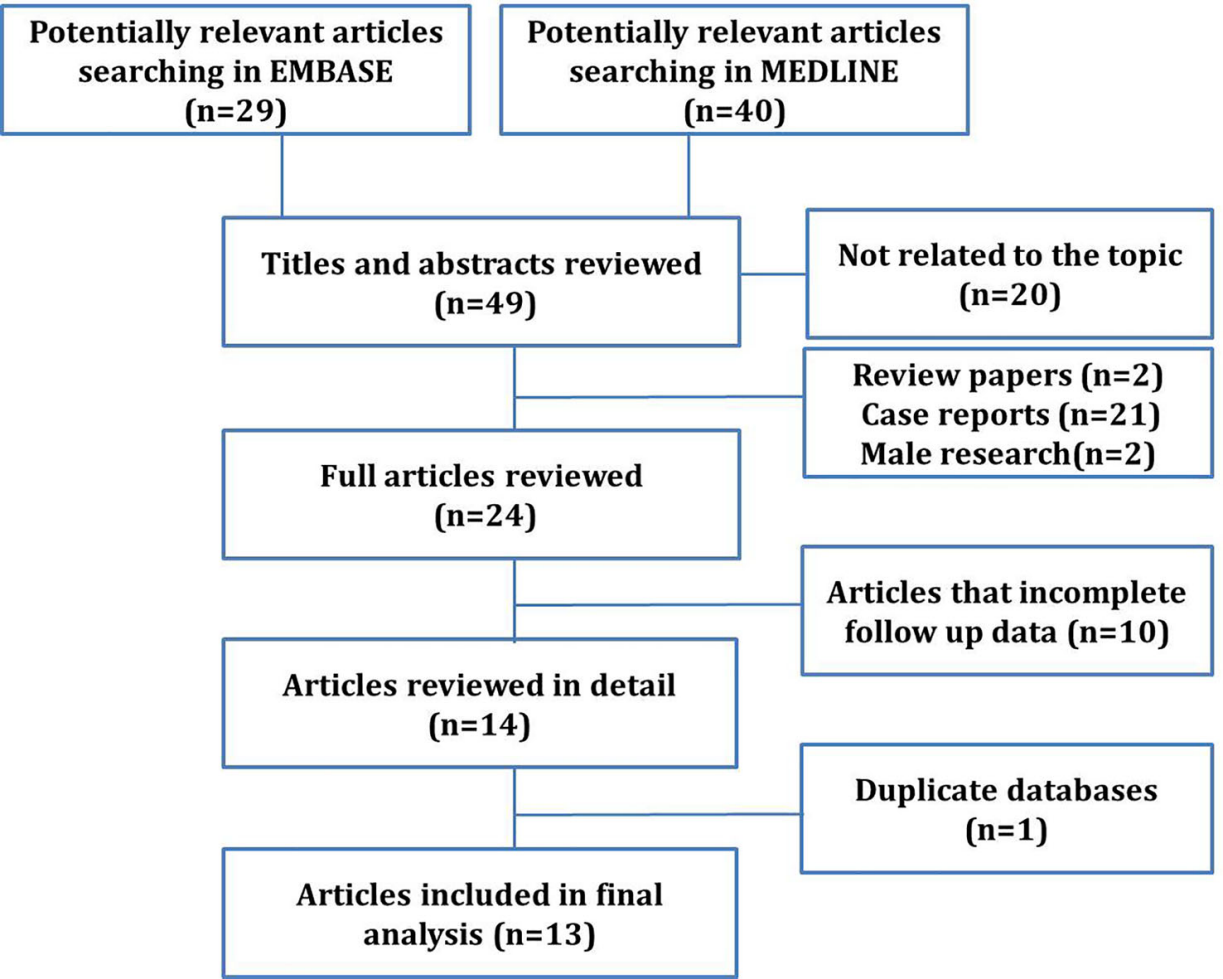
Flow diagram of literature search.

Abstracts obtained from electronic searches identified potentially relevant studies from year 2000 to February 2022 and were printed for analysis of significance (stage 1). Full-text articles were obtained for studies that were considered potentially relevant followed by screening to access full eligibility (stage 2). Literature search results and full-text articles that met full eligibility criteria were reviewed independently and in duplicate by two reviewers. Any disagreement was resolved through discussion with a third reviewer. Exclusion criteria included letters to the editor, case reports, reviews, articles that did not contain complete survival information, and non-English studies (**Figure 1**). Two series [Li-Ping Ge et al. (2018) and Byoung Hyuck Kim et al. (2017)] were similar articles from the Surveillance, Epidemiology, and End Results (SEER) database. Therefore, the articles with more comprehensive data were included.

### Outcome justification and prioritization

Our primary outcome was the mortality, and the secondary outcomes of interest included the distant metastasis and the local recurrences.

### Data extraction and quality assessment

The purpose of this meta-analysis was to analyze the status of surgery and RT in patients with OBC. Patient and study characteristics, intervention details, and outcomes of interest that met full eligibility were extracted.

The study characteristics recorded included the first author name, year of publication, study country of origin, the mean age of participants, and the mean follow-up in months (± SD). The patient characteristics recorded included the total number of patients investigated, lymph nodes status, hormone receptor status, HER-2 (human epidermal growth factor receptor 2) status, as well as the use of chemotherapy, radiation, and hormone therapy.

Surgical methods include modified radical mastectomy (MRM), breast-conserving surgery (BCS), and quadrantectomy or lumpectomy. All postoperative examinations confirmed that the breast lesions were benign. ALD dissection (ALND) included patients who were undergoing sentinel lymph node biopsy (SLNB) or standard ALND with preservation of the breast. Some patients received adjuvant RT, and the dose of RT was not recorded in detail in these studies. Most patients have received adjuvant chemotherapy or neoadjuvant chemotherapy. Unfortunately, these studies record neither the specific chemotherapy regimen nor the efficacy of neoadjuvant chemotherapy.

The patients included in this meta-analysis were classified into five groups: group 1, patients undergoing ALND alone; group 2, patients undergoing ALND with RT; group 3, patients undergoing ALND with BS (BS, including MRM or BCS or quadrantectomy or lumpectomy); group 4, patients undergoing ALND with RT and BS; and group 5, patients undergoing no surgery or RT alone. In this study, patients undergoing ALND included those who were undergoing SLNB or standard ALND with preservation of the breast.

### Statistical analysis

Statistical analysis was performed using Review Manager 5.1. (www.cochrane.org). Values are expressed as the mean ± standard deviation. The chi-square test (V2) was used to compare the characteristics of each group. P-values of <0.05 were considered statistically significant. The relative risk (RR) of the 95% confidence interval (CI) and the weighted aggregate estimation of the proportion of the 95% CI in the study control group are given. Statistical heterogeneity was measured using *I^2^* (*I^2^* > 50% was considered statistically significant heterogeneity). The average value was calculated to summarize the average value obtained from the study.

A random-effects model was used to aggregate data from the studies that were included in the meta-analysis. The random-effects model formally regards research heterogeneity as part of its goal. The use of heterogeneity in computational research is determined using the Cochran’s Q-statistical test, with a P-value of <0.1 indicating statistical significance. Cochran’s Q-statistical test allows for the evaluation of observed differences in results that are due only to chance. The degree of heterogeneity between studies was determined using I^2^ statistics, which estimated the proportion of effect changes caused by heterogeneity rather than by contingency. An I^2^ value of <25% represents low heterogeneity, of 25%–50% represents moderate heterogeneity, and of >50% represents significant heterogeneity. The quality of observational studies was assessed using the Newcastle–Ottawa quality assessment tool. A score of 0–9 was allocated to each observational study. Observational studies achieving scoring six to nine points were considered to be high quality, studies scoring four to five points were rated as moderate quality, and studies scoring three or fewer points were regarded as low quality (17).

## Results

### Clinicopathological characteristics of the included studies

Through a literature search, a total of 69 papers that were eligible for the study were selected, covering the period from 1990 to 2019. Through screening, 20 papers that not related to the topic were excluded. A total of 49 papers were reviewed thoroughly, and 25 articles were excluded because of case reports (21), summaries (1), nonsurgical comparative studies, searches with incomplete follow-up data (1), and male OBC studies (2). After excluding 10 articles with incomplete information and one duplicate article taken from the statistical data of the SEER database during a similar period, ultimately, there were 13 studies (18–30) met all the inclusion criteria and included in this meta-analysis (**Figure 1**).

A total of 3,476 patients were selected from 13 studies. All studies were retrospective cohort studies published between 2007 and 2021. In these studies, all patients underwent excisional or needle biopsies of the axillary mass, were confirmed to have metastasis from the breast, and were diagnosed with OBC by MRI or PET-CT before treatment (18–20). The median age and follow‐up in each study are shown in **Table 1**.

**Table 1:** Characteristics of studies included in the meta-analysis.

Study	Years of study	Country	median Age(y)	n	Preoperativeinspection	Follow-up (mo)	Study quality
Astrid Botty et al. (34)	2018-2019	USA	54	28	MRI	28^△^	4
Damian P. McCartan et al. (35)	1996-2001	USA	53	38	MRI	84(18-216)	7
Guiyun Sohn et al. (30)	1990-2009	Korea	47	142	--	78(15-198)	6
Haisong Yang et al. (36)	2010-2013	China	53	5	PET-CT	42 (9-72)	5
Lindsay K.Hessler et al. (37)	2004-2013	USA	--	1231	--	108(60-96)	6
Li-Ping Ge et al. (31)	2004-2014	Korea	--	394	--	60(4-151)	7
M. He et al. (29)	1998-2010	China	52	95	PET-CT	38.2(4-160)	7
Ramya Varadarajan et al. (38)	1997-2004	USA	58	10	MRI	57(16-84)	6
Sang Min Woo et al. (39)	1992-2010	Korea	47.5	40	MRI	71.5(5-205)	6
San-Gang Wu et al. (32)	1990-2013	China	--	980	--	53(60-120)	5
Yajing Huang et al. (40)	2005-2016	China	--	26	MRI/PET-CT	60(36-71)	7
Haeyoung Kim et al. (41)	2001-2013	Korea	54	53	MRI/PET-CT	85(7-178)	7
Catherine Tsai et al. (42)	2004-2014	USA	--	434	--	47.87^△^	5

--Detailed information was not mentioned

△Follow-up period was not mentioned

A total of 1,125 patients (632 with RT and 493 without RT) were treated with ALND or SLNB alone, and a total of 1,900 patients were treated with BS. The surgical patterns included the following: MRM, radical mastectomy, and BCS. Among the patients, 607 women aged less than 50 (17.5%) and 1,846 women aged more than 50 (53.1%); most patients’ lymph nodes status were N1 (1,885; 54.2%), N2 status (764; 22.0%), and least N3 (536; 15.4%); 1,587 estrogen receptor (ER)-positive tumors (45.7%), 1,366 ER-negative tumors (39.3%), 1,364 progesterone receptor (PR)-positive tumors (39.2%), and 1,483 PR-negative tumors (42.7%) were found; 288 HER-2–positive tumors (8.2%) and 452 HER-2–negative tumors (13.0%) were found, but HER-2 target therapy ratio was unknown. Chemotherapy was administered to 2,387 (68.7%) patients; among them, the rate of neoadjuvant chemotherapy was 32.4%, whereas that of adjuvant chemotherapy was 36.3%. Unfortunately, there is no detail of chemotherapy record. Endocrine therapy was administered to 1177 (33.9%) patients, and RT was administered to 2184 (62.8%) patients (**Table 2**).

**Table 2:** Patients’ characteristics.

Characteristic	N (value) 3,476		N (value)
Age		RT	2,184 (62.8)
<50	607 (17.5)	Done	1,184 (34.1)
>50	1,846 (53.1)	Not done	108 (3.1)
unknown	1,023 (29.4)	unknown	2,907 (83.6)
Number of positive LNs		Chemotherapy	2,387 (68.7)
≤3	1885 (54.2)	Neoadjuvant	1,126 (32.4)
4–9	764 (22.0)	Adjuvant	1,261 (36.3)
>9	536 (15.4)	Not done	569 (16.4)
unknown	291 (8.4)	Hormone therapy	
ER Status		Done	1,177 (33.9)
ER+	1,587 (45.7)	Not done	815 (23.4)
ER−	1,366 (39.3)	unknown	1,484 (42.7)
unknown	523 (15.0)	HER-2 status	
PR status		HER-2−	452 (13.0)
PR+	1,364 (39.2)	HER-2+	288 (8.2)
PR−	1,483 (42.7)	unknown	2,736 (78.8)
unknown	629 (18.1)		

LN, lymph nodes; ER, estrogen receptor; PR, progesterone receptor; RT, radiotherapy.

According to different surgical methods and treatment programs, we classified all patients into the following groups: group 1, patients undergoing ALND or SLNB only (493; 14.2%); group 2, patients undergoing ALND with RT (632; 18.2%); group 3, patients undergoing ALND with BS (1,483; 42.7%); group 4, patients undergoing ALND with RT and BS (467; 13.4%); and group 5, patients undergoing observation or RT only (401; 11.5%) (**Table 3**).

**Table 3:** Summary of the OS of included studies.

Study	n					
	ALND	ALND+RT	ALND+BS	ALND+RT+BS	Observation	RT only
Astrid Botty et al. (17)	20	0	0	0	8	0
Damian P. McCartan et al. (18) Guiyun Sohn et al. (19)	0 32	25 0	13 110	0 0	0 0	0 0
Haisong Yang et al. (20)	0	0	0	4	1	0
Lindsay K.Hessler et al. (21)	106	342	592	0	191	0
Li-Ping Ge et al. (23)	90	0	153	151	0	0
M. He et al. (24)	18	13	64	0	0	0
Ramya Varadarajan et al. (25)	8	0	2	0	0	0
Sang Min Woo et al. (26)	219	252	263	246	0	0
San-Gang Wu et al. (27)	0	0	29	0	0	11
Yajing Huang et al. (28)	0	0	8	18	0	0
Haeyoung Kim et al. (29)	0	0	5	48	0	0
Catherine Tsai et al. (30)	0	0	244	0	0	190
total	493	632	1483	467	200	201

ALND, axillary lymph node dissection; SLNB, sentinel lymph node biopsy; RT, radiotherapy; BS, breast surgery

**Figure 2:**
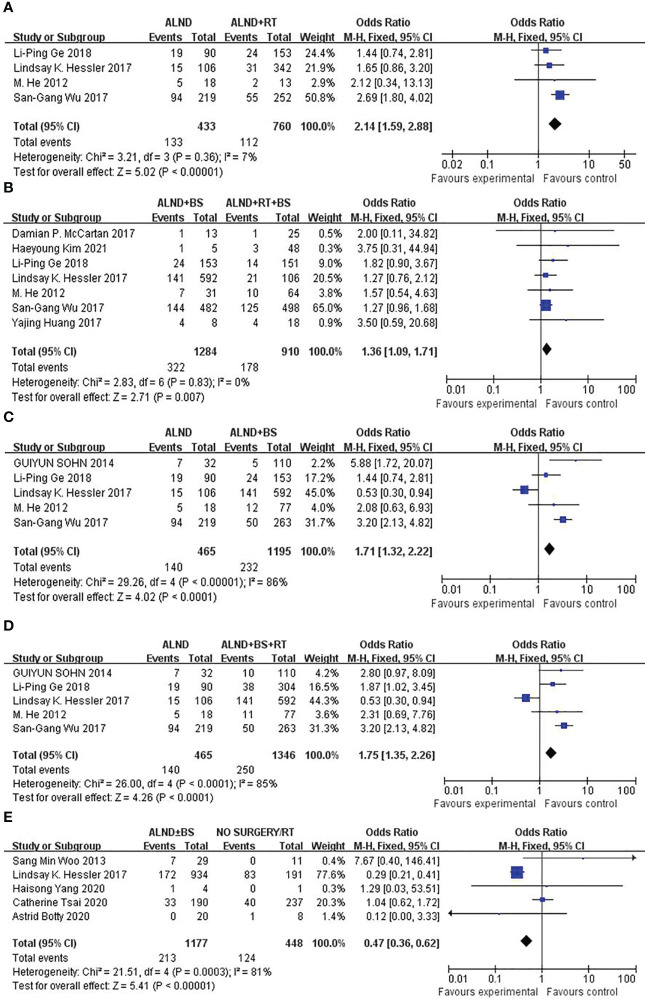
**(A)** Forest plot for mortality rates for patients undergoing ALND versus ALND + RT (p < 0.00001). **(B)** Forest plot for mortality rates for patients undergoing ALND + BS versus ALND + RT + BS (p = 0.007). **(C)** Forest plot for mortality rates for patients undergoing ALND versus ALND + BS (p < 0.0001). **(D)** Forest plot for mortality rates for patients undergoing ALND versus ALND + BS + RT (p < 0.0001). **(E)** Forest plot for mortality rates for patients undergoing ALND ± BS versus No surgery/RT (p < 0.00001).

To test the importance of RT in the prognosis of patients with OBC, we found that the mortality rates of patients undergoing ALND (group 1) and ALND + RT (group 2) were significantly different (30.7 vs. 14.7%, p < 0.00001; **Figure 2A**); the mortality rates of patients undergoing ALND + BS (group 3) and ALND + RT + BS (group 4) were also significantly different (25.1% vs. 19.6%, p = 0.007; **Figure 2B**). When evaluating the role of surgery in OBS treatment, the mortality rates of patients undergoing ALND (group 1) and ALND + BS (group 3) were also significantly different (30.7 vs. 19.4%, p < 0.0001; **Figure 2C**). Not unexpectedly, when comparing ALND (group 1) and ALND + RT + BS (group 4), group 4 also had an advantage in the mortality rate (30.7% vs. 18.6%, P < 0.0001; **Figure 2D**). Is there an advantage in survival between patients who have not undergone any surgery and patients who have undergone routine ALND ± BS surgery? We found that the mortality rate of ALND ± BS (groups 1 + 3) was more than that of no surgery or RT only (group 5) (21.4% vs. 31.0%, P < 0.00001; **Figure 2E**).

**Figure 3:**
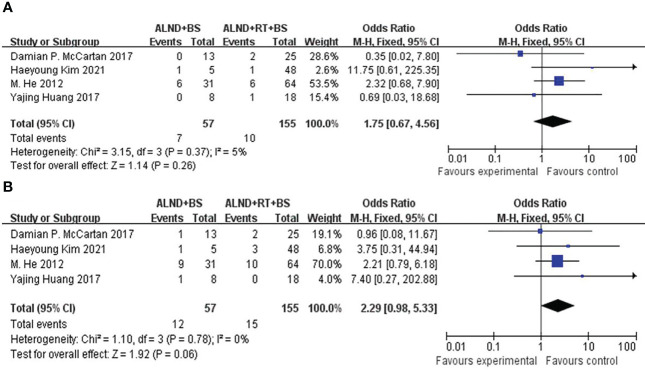
**(A)** Forest plot for distant recurrence for patients undergoing ALND + BS versus ALND + RT + BS (p = 0.06). **(B)** Forest plot for locoregional recurrence for patients undergoing ALND + BS versus ALND + RT + BS (p = 0.26).

DFS was rarely mentioned in these studies. To further inspect the prognosis of DFS, we have to merge groups to compare the survival differences of local recurrence and distant metastasis between ALND ± BS (groups 1 + 3) and ALND + RT ± BS (groups 2 + 4). We found that there was no significant difference both in the distant recurrence rates (21.0% vs. 9.7%, p = 0.06; **Figure 3A**) and locoregional rates (12.3% vs. 6.5%, p = 0.26; **Figure 3B**). However, the distant recurrence rate shows a significant trend of survival advantage.

### Risk of bias and quality assessment

**Figure 4:**
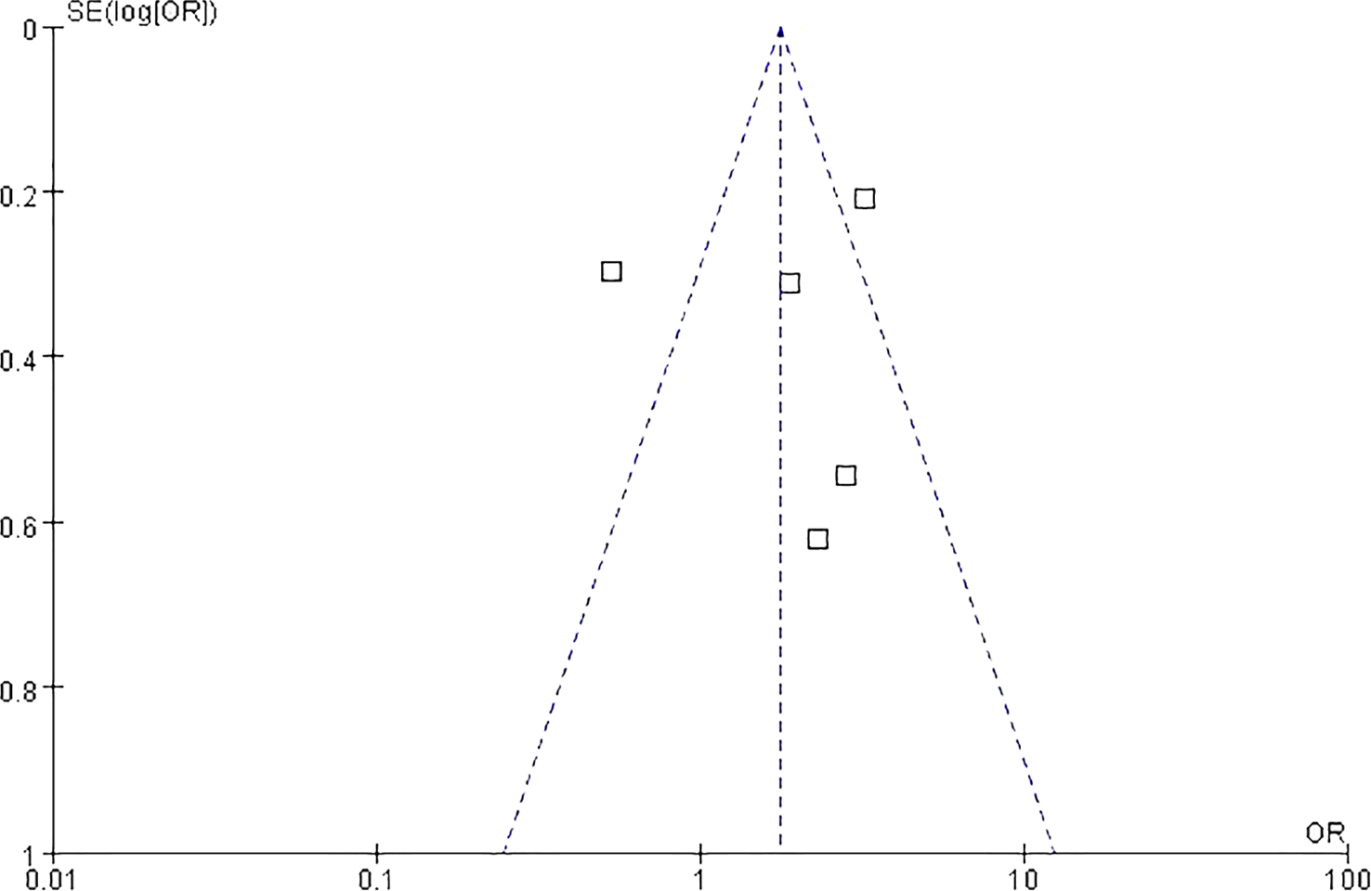
Funnel plot analysis on the detection of publication bias in the meta-analysis of prognostic significance.

Egger’s test was to assess publication bias. The funnel plot was approximately symmetrical and the result of Egger’s test (*P* = 0.67) revealed no obvious publication bias among the studies (**Figure 4**). Five (88.2%) meta-analyzed studies did not report median age of patients, and five (91.5%) meta-analyzed studies lacked any details imaging tests. Two (13.3%) meta-analyzed studies have not mentioned follow-up period time. Overall, the quality of evidence was poor, mainly because the included studies were retrospective studies—lack of prospective design, absence of study size calculation, and non-blinded assessment of results. The risk of bias across studies is summarized in.

## Discussion

This study is a meta-analysis with a more comprehensive analysis of surgical methods and a larger number of cases compared with past analyses of OBC. We provided an updated, more reliable conclusion on the comprehensive treatment with special breast cancer population (34). OBC has been conventionally considered a metastatic lymph node lesion from undetectable invasive breast cancer. Weaver et al. (4) found that occult metastases were an independent prognostic factor in patients with sentinel lymph nodes that were negative on initial examination. In the past, there was insufficient understanding because of the rarity of cases and the absence of large-scale randomized controlled trials. As we know, Z0011 study has led to significant changes in the surgical treatment of breast cancer, and the NCCN guidelines have recommend the surgery treatment of OBC; however, the optimal treatment for OBC is still controversial, and the key controversy is to accurately evaluate the appropriate mode of BS.

For systemic therapy, it is necessary to formulate chemotherapy, endocrine therapy, and targeted therapy according to the molecular type and clinical stage of patients. Because of the further understanding of pathogenesis, more signaling pathways have been found, and, correspondingly, therapeutic targets in breast cancer, such as hormone receptor, HER-2, epidermal growth factor receptor, and vascular endothelial growth factor, which optimize the accuracy of antitumor activity and minimize toxicity to normal tissues, play a crucial role in breast cancer treatment in the era of precision medicine (35, 36). Plenty of clinical trials and meta-analysis have found that chemotherapy and endocrine therapy can prolong survival of the subgroups of ER- and PR-positive patients (37, 38).

For local treatment, the mode of breast surgery and theaxillary lymph node management are the main controversial points. In all the included retrospective studies, total mastectomy with ALND or whole-breast RT + ALND seemed to perform more common treatment strategies. However, the prognosis varies greatly between groups (27, 39).

In our study, we included information about OBC in the SEER database from 1990 to 2019, which, to the best of our knowledge, is the largest data available at present (23, 26). Some studies suggested that BS does not improve the prognosis of patients with OBC with only ALD disease, whereas Rueth et al. (40) considered that RT should be used for N1 patients with ALD metastasis. Therefore, BS does not improve the prognosis of patients with OBC. Among the cases that we collected, patients who underwent BCS or total mastectomy were confirmed to have benign lesions by postoperative pathology. We classified these cases into one category compared with patients without BS. Through the comprehensive comparison of different treatments, we obtained more comprehensive and objective information. We found that BS, including biopsy of suspected lesions and total mastectomy, can improve the prognosis of patients, suggesting that some truly “occult” lesions that cannot be found by pathological examination can be removed by surgery, which was different from the results of some studies.

Our results show that both increased RT and BS, compared with simple ALND, can improve the prognosis of patients. Whether based on ALND or BS, RT can further improve OS. This is consistent with most of the study results (22, 41, 42). Unfortunately, there was no specific description of the RT dose in the included studies. Moreover, some studies reported that the prognosis of breast + axillary RT is similar to that of surgery, and the choice of local RT can replace surgery, which is controversial in the conclusions of these studies (4, 30). Because of the lack of evaluable primary lesions, there were also great differences in the patterns of BS. Some studies have attempted to take BCS and benign lesion surgery as the research objectives and found that BCS combined with RT was an effective treatment (16, 32).

Patients who received neoadjuvant chemotherapy have equal survival after breast-conserving therapy compared with mastectomy and appeared to have better survival than patients undergoing mastectomy without radiation (43, 44), suggesting that patients with OBC receiving neoadjuvant chemotherapy may benefit from survival. From our results, we confirmed that increasing BS, including MRM and BCS, can increase the survival of patients (29). This shows that, although patients with OBC do not have definite lesions in the breast, increasing the powerful local control of the breast, despite the use of surgery or regional RT, can significantly improve the survival time of these patients (45).

Previous studies reported that breast + axillary RT has the same prognosis as surgical treatment, and local RT can also be effective for patients with OBC. These research conclusions are controversial (31, 33). Our meta-analysis showed that ALND + BS can significantly improve the prognosis of patients compared with RT only or observation. In addition, RT can undoubtedly improve the survival of patients with OBC. For N1 patients, SLNB combined with RT may be an alternative to ALND. Some research studies show that SLNB may be an option in selected patients with OBC who downstage following neochemotherapy (46). Our analysis found that, for patients with ALND only or ALND + BS, RT can significantly improve their OS. This is consistent with most research results (21, 24). As the number of lymph node metastases is an independent prognostic factor, RT may further control local recurrence (23). However, from our analysis, this benefit is not shown for the DFS aspect. Compared with ALND + BS, ALND + BS + RT cannot prolong both the time of distant metastasis and local recurrence, but the distant recurrence rate shows a significant trend of survival advantage, suggesting that local recurrence may be controlled by RT, but RT has no effect on OS. With the continuous improvement of comprehensive treatment, patients with OBC treated with neoadjuvant therapy are generally considered to have a better prognosis than breast cancer patients who do not receive this treatment (25, 47–49). In the case of notable effects of systemic comprehensive treatment, SLNB + RT may be considered an alternative to ALND in patients with breast cancer.

We explored the possibility of more nonsurgical treatment without reducing the treatment effect. Our meta-analysis confirmed that, although there may be undetectable lesions in the breast, BS and RT can further improve the survival and prognosis of patients, which further provides a reliable basis for the standardized treatment of OBC. We acknowledge that there are some limitations in this study, as the studies we included were nonrandomized retrospective studies, thus limiting the quality of available data in the literature. Moreover, patients included in this study may have been subjected to variable doses of radiation, different hormone levels, chemotherapeutic agents, and hormonal therapy.

## Conclusions

It indicates that surgery including MRM or BCS + RT is the best strategy for breast cancer treatment according to our results. RT cannot prolong both the time of distant metastasis and the local recurrences. Further research to be explored is the effect on decision-making in surgery strategy.

## Data availability statement

The raw data supporting the conclusions of this article will be made available by the authors, without undue reservation.

## Author contributions

RW, JJH, JC, and QL designed the study. RW, HXY, and JC contributed to analysis and interpretation of the data. RW and HXY were major contributors in writing the manuscript. All authors contributed to the article and approved the submitted version.
